# The Western Aortic Collaborative: Multi-institutional Aortic Surgery, Education, and Research

**DOI:** 10.1055/s-0045-1809344

**Published:** 2025-05-29

**Authors:** Fenton H. McCarthy, Christopher R. Burke, Jason P. Glotzbach, Michael P. Fischbein, Anthony Caffarelli, Fernando Fleischman, T. Brett Reece

**Affiliations:** 1 Department of Cardiothoracic Surgery, Providence Heart Institute, Sacred Heart Medical Center, Spokane, Washington; 2 Department of Cardiothoracic Surgery, University of Washington, Seattle, Washington; 3 Department of Cardiothoracic Surgery, University of Utah, Salt Lake City, Utah; 4 Department of Cardiothoracic Surgery, Stanford, Palo Alto, California; 5 Department of Cardiothoracic Surgery, International Heart Institute, St. Patrick's Hospital, Missoula, Montana; 6 Cardiothoracic Surgery, University of Southern California, Los Angeles, California; 7 Cardiothoracic Surgery, University of Colorado, Aurora, Colorado

**Keywords:** thoracic aortic surgery, clinical research, aortic dissection, aortic valve repair, aortic arch

## Abstract

**Objective:**

The Western Aortic Collaborative (WAC) is a new, hybrid, multi-institutional academic model that seeks to perform aortic surgery research and education.

**Methods:**

The WAC has three fundamental thoracic aortic surgery goals: (1) advancing surgical techniques, (2) furthering education and development of aortic surgeons, and (3) performing multi-institutional clinical research. The WAC utilizes a hybrid model of annual in-person meetings at the Western Thoracic Surgical Association (WTSA) combined with videoconferencing platforms that regularly connect surgeons dispersed throughout the Western region. The structure of WAC is intentionally horizontal. The goal is to promote cross-pollination of ideas, techniques, and experiences between surgeons at different institutions.

**Results:**

For its research goals, the WAC first identified the principal areas of aortic surgery with the greatest knowledge gaps and which of those knowledge gaps could best be addressed by institutional practice differences within WAC. Using this natural experiment design, five high-priority research topics from the aortic root to the left subclavian artery were created. In order to perform this subspecialized, multi-institutional research, the WAC created a novel, cloud-based database that piggybacks on the Society for Thoracic Surgeon database. The combined database also preserves the ability to generate subspecialized variables and to link with each institution's medical record system for semi-automated functionality.

**Conclusion:**

In its inaugural year, the WAC succeeded in its primary goals of utilizing remote technology platforms and the annual WTSA meeting to create a regional community of aortic surgeons with shared research and educational goals.

## Introduction

Surgeons are routinely “siloed” within their health systems. With a relatively high concentration of aortic surgery, research, and training within a limited number of institutions, aortic surgeons have historically exchanged ideas through publication of original research or intermittent collaboration at scientific meetings. A more dynamic method of collaboration and sharing of ideas would likely increase dissemination of knowledge within cardiothoracic surgery.

The Western Aortic Collaborative (WAC) is a new, hybrid, multi-institutional academic model that seeks to address the need for more dynamic professional collaboration by leveraging multimedia platforms and communication technology.

## Materials and Methods

The study was approved by the IRB at the University of Colorado COMIRB# 22-2254 on January 20, 2023. The WAC has three fundamental thoracic aortic surgery goals: (1) advancing surgical techniques, (2) furthering education and development of aortic surgeons, and (3) performing multi-institutional clinical research. The WAC utilizes a hybrid model of annual in-person meetings at the Western Thoracic Surgical Association (WTSA) combined with newer communication technology platforms that regularly connect surgeons dispersed throughout the Western region. The structure of WAC is intentionally horizontal, and the goal is to promote as much cross-pollination of ideas, techniques, and experiences between surgeons at different institutions as possible.

The collaborative approach to the WAC started with the pursuit of like-minded aortic surgeons interested in advancing aortic surgical techniques. The hybrid model of the WAC includes structured biweekly videoconferencing meetings that start with specific case questions and recent lessons learned in the field of aortic surgery from group members. The diversity of the group in terms of aortic surgical training, current practice environment, and surgical techniques promotes a high-level discussion. Future cases and best practices for adopting new technology are discussed as well.

For the education goals, the WAC has two primary goals: (1) peer mentoring, development and education; (2) resident and fellow education and training. The regular interactions of WAC members through online meetings and the annual WTSA provide the structure for the peer education and mentoring. The WAC has a similar hybrid approach to resident and fellow education. In-person meetings will be used to provide education sessions for trainees. The WAC will also have a public online presence in the form of webinars, which will be memorialized on a website along with surgical technique videos.

The third pillar of the WAC is multi-institutional research along with peer-reviewed publications. WAC members first identified the areas of greatest aortic surgery uncertainty or equipoise for potential collaborative research products. WAC members systematically went through the possible research topics to identify areas of interest with individual members as well as those matching a natural study design based on practice differences between institutions within the WAC. The results of these studies will be submitted to peer-reviewed publications.

An adequate database and research infrastructure to perform the studies designed by the WAC did not exist. A new, multi-institutional database with the capability to link with individual institutions in a HIPAA-compliant manner is being created through REDCap Cloud. The database is built off of the Society for Thoracic Surgeon (STS) database variables with additional specific preoperative, operative, postoperative, and follow-up variables created to answer tailored aortic surgery research questions. The database is designed to link with multiple different types of institutional electronic medical records.

## Results

**Fig. 1:**
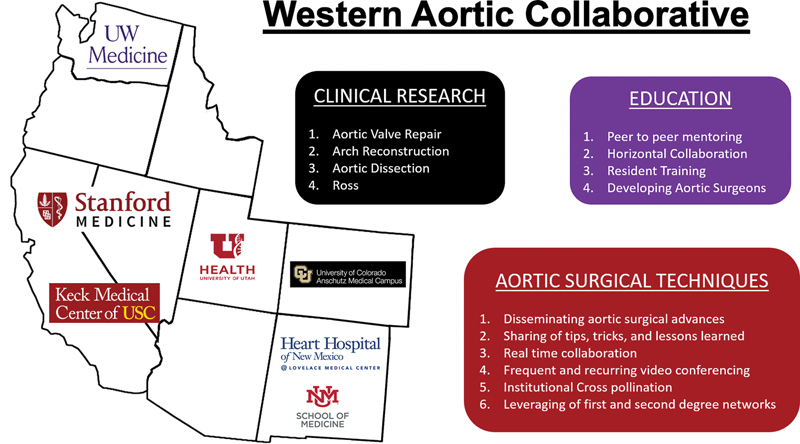
The institutions and primary goals of the Western Aortic Collaborative.

**Fig. 2:**
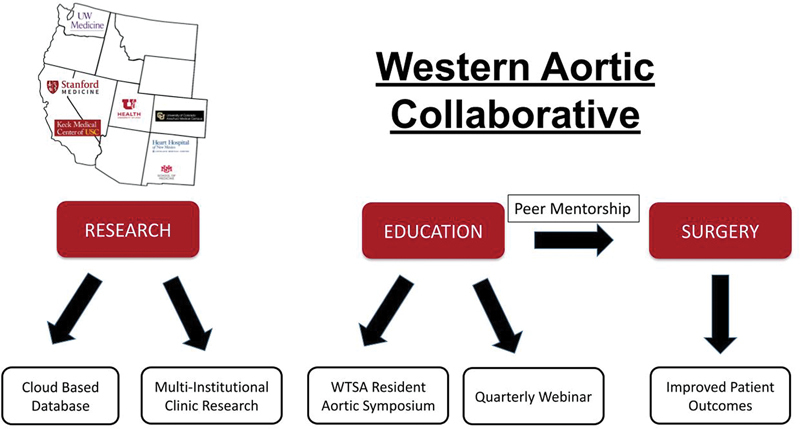
Aortic surgeons and methods of the Western Aortic Collaborative. WTSA, Western Thoracic Surgical Association.

A group of aortic surgeons gathered at the WTSA 2022 annual meeting and discovered they shared the same goals to pursue multi-institutional research and disseminate aortic surgery techniques. There was also a shared sense of urgency to change the pace and methods with which these goals were achieved. Fig. 1 shows the geographic regions and primary goals of the original WAC institutions. Fig. 2 shows the strategic plan and methods to achieve the WAC goals of research, education, and advancing aortic surgical techniques.

For its research, the WAC principal areas of aortic surgery identified by the WAC were aortic valve repair techniques, Ross procedure techniques and outcomes, management of the aortic arch in acute aortic dissection, management of the left-subclavian artery during aortic surgery, and temperature and cannulation techniques in elective aortic surgery.

In order to perform this subspecialized, multi-institutional research, the WAC is creating a novel cloud-based database that piggybacks on the STS database. The combined database also preserved the ability to generate subspecialized variables and to link with each institution's medical record system for semi-automated functionality. The database and primary research goals will take time to accrue patients and complete the analysis. In the meantime, the WAC is focused on specific questions and vexing situations facing aortic surgeons.

For its mission of disseminating aortic surgical techniques and peer mentoring, the biweekly videoconferencing meetings, a group text platform, and annual in-person meetings at the WTSA have allowed WAC aortic surgeons to solicit expert opinion from other surgeons. The hybrid platform in particular offers an ideal way to share lessons learned in rare and vexing situations or seek advice on future cases, which have then been implemented as part of real surgical plans. Some specific examples included ascending to descending aortic bypass, internal fixed ring annuloplasty for aortic valve repair, and management of infected thoracic endovascular aortic repair (TEVAR) grafts.

The educational arm of WAC was introduced at the 2023 WTSA annual meeting with a special discussion section on management of aortic dissection. The education from the WAC is broadly structured to be relevant to surgeons at every stage of their career from trainee to senior surgeon and society leader. The future plans include regular webinars focused on specific areas of aortic surgery and to memorialize these webinars on a website along with accompanying technical surgical videos.

## Discussion

The WAC is composed of aortic surgeons within the Western United States who share the overarching goal to improve the quality of aortic surgery, as it is performed, taught, and investigated. The primary goals of the WAC have precedents in previous quality improvement and research endeavors in cardiothoracic surgery and aortic diseases, but they are also distinct and novel from these previous efforts.

Some of the most successful efforts to improve health care delivery and perform practice changing research have occurred in surgery and in cardiothoracic surgery specifically. Based on the success of surgical research performed at the Veterans Affairs Hospitals in the 1980s and early 1990s, the American College of Surgeons created the National Surgical Quality Improvement Program (NSQIP). 1 With more than 100,000 major surgical cases each year, NSQIP has been associated with significant improvements in surgical outcomes with a 47% decrease in surgical mortality from 1991 to 2006. 2

Within cardiothoracic surgery, there has been collaboration between surgeons and institutions for data-driven quality improvement both at the national and the regional level. The STS National Database was established in 1989 to track cardiothoracic surgical outcomes, perform risk adjustments, and improve outcomes. Widely regarded as one of the most successful and impactful clinical databases, the STS National Database includes more than 8 million patients and 4,300 surgeons and has generated associated databases in Congenital Heart Surgery and Thoracic Surgery. 3 The importance and utility of the STS database was validated as it became part of the STS and American College of Cardiology (ACC) Transcatheter Valve Therapies Registry (STS/ACC TVT). The national coverage decision by the Centers for Medicare and Medicaid Services for new transcatheter therapies, including transcatheter aortic valve replacement and transcatheter edge-to-edge repair, includes participation in the STS/ACC TVT Registry. 4

For the purposes of the WAC, new technology adoption is part of all three of its major goals including advancing surgical techniques, outcomes research, education, and mentoring. There has been an explosion of new aortic surgical technologies in recent years including internal aortic valve annuloplasty rings, branched TEVAR grafts, uncovered dissection stents, hybrid surgical-TEVAR grafts, and others. Not every major aortic center is part of the clinical trial for each device, and all of the successful trial technologies eventually move on to implementation in real world settings. Part of the WAC's efforts in education and mentoring is sharing experiences with new technologies as they are rolled out and strategically planning future comparison studies based on different practice patterns at different WAC institutions.

While the national registries such as the STS/ACC TVT Registry and others have improved patient outcomes, informed health policy, and tracked new technologies; there is still a need to meet regional research and outcomes goals within cardiothoracic and aortic surgery. There are examples of these initiatives outside of the WAC. The Virginia Cardiac Services Quality Initiative (VCSQI) is a statewide initiative that seeks to improve outcomes in cardiothoracic surgery with a concerted effort towards “peer-to-peer value exchange.” 5 6 The WAC shares significant common ground with VCSQI in its scope, mission, and scale. The WAC differs from VCSQI in that it spans a far larger geographic region.

In order for the WAC to build peer relationships and further its research goals across its wide geographic region, the WAC uses a hybrid model that intentionally incorporates modern technology platforms. The WAC has biweekly videoconferencing as well as HIPAA-compliant text communications. This setup maintains regular research and education meetings as well as real-time communication between members spread across thousands of miles in the Western United States. This structure has resulted in an immediate impact on participating surgeon practices, case planning, and patient outcomes. It has also accelerated the complex process of carrying out multi-institutional research based off of a newly created cloud based database linking different institutional and different types of electronic medical records.

From a disease-based perspective as opposed to geographically or specialty-based organization, there are a number of active multi-institutional groups within the fields of aortic disease diagnoses and therapies. The GenTAC Alliance was founded in 2017 and based off of the National Registry of Genetically Triggered Thoracic Aortic Aneurysms and Related Cardiovascular Conditions. The goals of GenTAC include research, registry partnerships, and clinical management of thoracic aortic diseases. 7 There is also the international organization the Montalcino Aortic Consortium, which focuses on the study of variations in genes affecting the thoracic aorta. 8

The multi-institutional collaborations based around the surgical therapies for thoracic aortic disease include the International Aortic Arch Surgery Study Group (IAASSG), and the International Registry of Acute Aortic Dissection (IRAD). 9 10 The WAC shares the focus on aortic surgical outcomes research with IRAD and IAASSG, but the WAC approach differs in its effort to identify current areas of surgical equipoise or technical challenges. The research of the WAC is geared towards informing specific questions routinely faced by aortic surgeons. Topics include management of left subclavian artery in aortic surgery; standard versus conditional approaches to hemiarch versus total arch replacements in acute aortic dissections; and cannulation and temperature management in elective aortic surgery. While these areas have been previously researched to varying degrees, these studies are often statistically underpowered, tied to the experience and/or capabilities of a single institution, or lack details necessary to change the practice of aortic surgery.

The WAC differs from previous aortic collaborations in its combined goals of education, peer mentoring, and adoption of new technology. The WAC seeks to provide aortic surgical education and mentoring for all phases of an aortic surgeon's career. The WAC also recognizes and seeks to address the needs of practicing aortic surgeons interested in comparing and perfecting techniques outside the confines of one institution or networks of similar training background. The WAC values the diversity in approaches and the cross-pollination of ideas in aortic surgery as a unique contribution towards advancing the field.

## Conclusion

There is a recognized need to perform collaborative aortic surgical- and disease-based research. The WAC is a newly formed group of aortic surgeons with the intent of regional collaboration around the shared goals of aortic surgery clinical research, education, mentoring, and advancing surgical techniques. The WAC uses a hybrid meeting structure and a multi-institutional aortic surgery database in order to address specific questions and challenges currently facing aortic surgeons.
